# NfiS, a species-specific regulatory noncoding RNA of *Pseudomonas stutzeri*, enhances oxidative stress tolerance in *Escherichia coli*

**DOI:** 10.1186/s13568-019-0881-7

**Published:** 2019-09-25

**Authors:** Guihua Hu, Tao Hu, Yuhua Zhan, Wei Lu, Min Lin, Yunhong Huang, Yongliang Yan

**Affiliations:** 1 College of Life Sciences, Jiangxi Normal University, Nanchang, 330022 People’s Republic of China; 2 Biotechnology Research Institute, Chinese Academy of Agricultural Sciences, Beijing, 100081 People’s Republic of China

**Keywords:** *Pseudomonas stutzeri* A1501, NfiS, Recombinant *Escherichia coli*, Oxidative stress, Regulatory element

## Abstract

Noncoding RNAs (ncRNAs) can finely control the expression of target genes at the posttranscriptional level in prokaryotes. Regulatory small RNAs (sRNAs) designed to control target gene expression for applications in metabolic engineering and synthetic biology have been successfully developed and used. However, the effect on the heterologous expression of species- or strain-specific ncRNAs in other bacterial strains remains poorly understood. In this work, a *Pseudomonas stutzeri* species-specific regulatory ncRNA, NfiS, which has been shown to play an important role in the response to oxidative stress as well as osmotic stress in *P. stutzeri* A1501, was cloned and transferred to the *Escherichia coli* strain Trans10. Recombinant NfiS-expressing *E. coli*, namely, Trans10-*nfiS*, exhibited significant enhancement of tolerance to oxidative stress. To map the possible gene regulatory networks mediated by NfiS in *E. coli* under oxidative stress, a microarray assay was performed to delineate the transcriptomic differences between Trans10-*nfiS* and wild-type *E. coli* under H_2_O_2_ shock treatment conditions. In all, 1184 genes were found to be significantly altered, and these genes were divided into mainly five functional categories: stress response, regulation, metabolism related, transport or membrane protein and unknown function. Our results suggest that the *P. stutzeri* species-specific ncRNA NfiS acts as a regulator that integrates adaptation to H_2_O_2_ with other cellular stress responses and helps protect *E. coli* cells against oxidative damage.

## Introduction

By base pairing with target sequences within mRNA or proteins, noncoding RNAs (ncRNAs) in bacteria, also known as small RNAs (sRNAs), can modulate gene expression mainly at the posttranscriptional level (Wassarman et al. 1999). The macromolecules that interact with sRNAs were found to be involved in the regulation of a variety of biological processes, such as stress response, virulence, motility, biofilm formation, nitrogen fixation, quorum sensing, and metabolic control (Lenz et al. 2005; Nakamura et al. 2007; Romby et al. 2006; Skippington and Ragan 2012; Yuan et al. 2015; Zhan et al. 2016).

In the processes of bacterial growth and development, Reactive oxygen species (ROS), such as superoxide anion radical (O_2_^–^), hydrogen peroxide (H_2_O_2_) and hydroxyl radicals (·OH), are generated continuously (Blokhina et al. 2003; Imlay 2008). Consequently, living organisms, with enzymes such as catalase and superoxide dismutase (SOD), proteins such as thioredoxin and glutaredoxin, and molecules such as glutathione (Trotter and Grant 2003), have evolved defense mechanisms and genetic responses that limit oxidative stress by detoxifying ROS, including H_2_O_2_.

Because of its clear genetic background, *Escherichia coli* has been used as a model strain for scientific research and is a widely used strain in industrial production (Liang et al. 2011; Negrete and Shiloach 2017). However, facultative aerobic growth of organisms to help them acquire the ability to protect themselves against oxidative stress has been a challenge for the biotechnological industry, especially for the production of recombinant proteins.

OxyR, SoxRS and RpoS are three major regulators known to function as homeostasis-maintaining component in the context of cellular oxidative stress by acting as transcriptional regulators of several genes (Chiang and Schellhorn 2012). In addition, some molecules are constitutively present to scavenge chemically reactive oxygen or help maintain a reducing intracellular environment. For example, Fe^2+^ play a significant role in activation of molecular oxygen, reduction of ribonucleotides, decomposition of peroxides and electron transport, thereby protecting the cell or organism from oxidative stress (McHugh et al. 2003).

Recently, using synthetic sRNAs that was based on the abilities and characteristics of natural sRNAs led to the identification of a level of RNA-mediated regulation, greatly contributing to our understanding of the multiple levels of control used by cells and the interactions among these control mechanisms (Na et al. 2013; Yoo et al. 2013). In addition to the antioxidant ncRNA OxyS of *E. coli* (González-Flecha and Demple 1999), some ncRNAs act as positive regulators in *Pseudomonas* spp. to provide resistance to oxidative stress. In *Pseudomonas fluorescens* and *Pseudomonas syringae*, the ncRNA RgsA has been reported to be important for resistance to oxidative stress (Gonzalez et al. 2008; Park et al. 2013). In the nitrogen-fixing strain *Pseudomonas stutzeri* A1501, the function of an ncRNA NfiS is related to the stress response; in the presence of 20 mM H_2_O_2_ or 0.3 M sorbitol, the *nfiS* mutant was more sensitive than the wild-type (WT) *P. stutzeri* A1501, whereas overexpression of NfiS led to enhanced resistance. Thus, NfiS plays an important role in the response to oxidative or osmotic stress, and NfiS likely contains another as-yet-unidentified nucleotide sequence that pairs with stress resistance-related target genes (Zhan et al. 2016).

To investigate whether NfiS has a similar function in *E. coli*, *nfiS* (NfiS-encoding gene) from *Pseudomonas stutzeri* A1501 was cloned into the mobilizable vector pLAFR3 and transformed into the *E. coli* strain Trans10 to obtain recombinant strain Trans10-*nfiS*, and Trans10-pLAFR3 (Trans10 harboring the plasmid pLAFR3) was constructed as a control. The WT *E. coli* and the recombinant strains were treated with 20 mM H_2_O_2_ for 10 min or shocked with 1.5 M NaCl for 60 min. The recombinant strains exhibited stronger resistance to H_2_O_2_ and osmotic stress than the WT *E. coli*. These results indicate that NfiS also plays a similar role in *E. coli*. Here, we report that an ncRNA from *P. stutzeri* A1501, namely, NfiS, improved the oxidative and osmotic stress resistance of *E. coli.* Further microarray analysis showed that NfiS influenced gene expression in *E. coli*, allowing investigation of the specific molecular mechanism underlying the improvement of oxidative and osmotic stress resistance in *E. coli* by NfiS.

## Materials and methods

### Bacterial strains and plasmids

The strains and plasmids are listed in Table 1. *P. stutzeri* A1501 was grown in minimal lactate medium or in Luria–Bertani (LB) medium at 30 °C as previously described (Desnoues et al. 2003). *E. coli* and derivatives were grown in LB medium at 37 °C. When appropriate, media were supplemented with 10 µg/mL antibiotic tetracycline (Tc).

**Table 1:** Strains and plasmids used in this study

Strains and plasmids	Relevant characteristics	Source or reference
*P. stutzeri*		
A1501	WT, Chinese Culture Collection: CGMCC 0351	Desnoues et al. (2003)
*E. coli*		
Trans10	*Escherichia coli* competent cells	TransGen Biotech, Beijing, China
Trans10-pLAFR3	Trans10 containing pLAFR3, Tc^r^	This study
Trans10-*nfiS*	Trans10 containing pLA-*nfiS*, Tc^r^	This study
Plasmids		
pLAFR3	Mobilizable vector, Tc^r^	Staskawicz et al. (1987)
pLA-*nfiS*	pLAFR3 derivative carrying the WT A1501 *nfiS* gene under the control of its endogenous promoter, Tc^r^	Zhan et al. (2016)

### Cloning of the *nfiS* gene from *P. stutzeri* A1501

The complete genome of *P. stutzeri* A1501 was deposited in the GenBank database with accession no. CP000304 (Yan et al. 2008). The complete *nfiS* gene amplified from A1501 genomic DNA was ligated into the plasmid pLAFR3, and then the resulting plasmid pLA-*nfiS* was transformed into *E. coli* Trans10 cells to generate the strain Trans10-*nfiS*. We next assayed if the identical size of NfiS (254 bp) was conferred to *E. coli* and expressed by reverse-transcription PCR (RT-PCR). Total RNA from Trans 10 and Trans 10-*nfiS* was extracted and converted into cDNA via reverse transcription (PrimeScript™ RT reagent Kit, Takara, Japan) according to the manufacturer’s protocol. Synthesized cDNA samples were amplified by using primers *nfiS*-F (5′-CCGCTGTCTGGCCTGTT-3′) and *nfiS*-R (5′-CCATGGGTGCCCGAATC-3′).

### Growth rate and culture conditions

For the growth assay, cells from an overnight culture in LB medium were centrifuged and resuspended in 65-mL flasks containing 10 mL of LB medium at an OD_600_ of 0.1, and then 400 μL bacterial suspensions were incubated in the automatic growth curve analyzer Bioscreen C FP-1100-C (OY Growth Curves AB Ltd, Finland) at 37 °C. Culture samples were taken every 20 min, and the cell density was determined spectrophotometrically at 600 nm (OD_600_).

### H_2_O_2_ and NaCl shock treatments

For H_2_O_2_ shock treatments, *E. coli* strains were cultured overnight and transferred into fresh LB broth the next day until the OD_600_ increased to 0.6. Then, 20 mM H_2_O_2_ was added to the medium, and the culture was incubated at 30 °C and 220 rpm for 10 min. Next, 10 serial dilutions were prepared for all the strains, and 8 µL of each dilution was spotted onto LB agar plates. The plates were incubated at 30 °C for 16 h.

For NaCl shock treatments, *E. coli* strains were cultured overnight and were transferred into fresh LB broth the next day until the OD_600_ increased to 0.6. Then, 1 mL of the suspension was collected, and the cells were resuspended with 1 mL of 1.5 M NaCl and incubated at 30 °C and 220 rpm for 1 h. Then, 10 serial dilutions were prepared for all strains, and 8 µL of each dilution was spotted onto LB agar plates. The plates were incubated at 30 °C for 16 h.

### Genome-wide cDNA microarray analysis

Both WT *E. coli* Trans10 and Trans10-*nfiS* samples after H_2_O_2_ shock for 10 min were collected (see H_2_O_2_ shock treatment in “Materials and methods”). Total RNA samples were extracted from three independent experiments of the Trans10 and Trans10-*nfiS* strains. Isolation of RNA from *E. coli* was carried out by using a combination of TRIzol reagent and the Qiagen RNeasy Mini Kit with DNase I treatment (Invitrogen, USA). The concentration and purity of the RNA were evaluated using a NanoDrop ND-1000 spectrophotometer (Thermo Scientific, USA) followed by 1.2% formaldehyde gel electrophoresis. cDNA was synthesized from 10 µg of total RNA using One-Cycle Target Labeling and Control Reagents (Affymetrix, USA) to produce biotin-labeled cDNA. The quality and quantity of the original RNA samples and the cDNA probes generated for array hybridization were determined with a NanoDrop ND-1000 spectrophotometer. Following fragmentation, 10 µg of cRNA was hybridized for 16 h at 45 °C on GeneChip *Escherichia coli* Genome Arrays. GeneChips were washed and stained in an Affymetrix Fluidics Station 450 and scanned using the Scanner 3000 7G 4C system. The data were analyzed with Microarray Suite version 5.0 (MAS 5.0) using Affymetrix default analysis settings and global scaling as a normalization method. The trimmed mean target intensity of each array was arbitrarily set to 100. Log2 ratio > 1.0 or < − 1.0 was considered to be significantly different. Information for each probe was obtained according to Gene Ontology (GO) classification, and the genes were annotated for classification of functional and biological processes.

### Quantitative real-time PCR

Total RNA was isolated using the innuPREP RNA Mini Kit (Analytik jena, Germany). For each sample, 1.5 μg of the total RNA of three independent biological replicates was reverse transcribed using a PrimeScript™ RT Reagent Kit with gDNA Eraser (Perfect Real Time) (TaKaRa, Japan) following the procedure in the manual. HiScript^®^ II Q RT SuperMix for qPCR (Vazyme, China) was used to remove genomic DNA and generate first-strand cDNA. The produced cDNA was used to perform qRT-PCR using ChamQ™ Universal SYBR qPCR Master Mix. The reactions were performed using the 7500 sequence detection system (Applied Biosystems, USA), and the relative expression of the genes was calculated using the 2^−ΔΔCt^ method. In this experiment, 16S rRNA was used as an internal standard, and data analysis was carried out according to the manufacturer’s recommendations. Primer information is shown in Additional file 1: Table S1.

### Prediction of the NfiS RNA target genes in *E. coli*

The target genes of ncRNA NfiS (254 bp) were predicted by using IntaRNA (Busch et al. 2008). The hybridization between the sRNA transcript sequence and the sequence comprising 300 nt upstream and 300 nt downstream of the start codon of each annotated gene was screened in the genome of *Escherichia coli* str. K-12 substr. MG1655. GO annotations with the default parameters and GO enrichment with EASE scores of 0.05 were determined with the functional annotation tool DAVID. Based on hybridization energy and accessibility of the interaction sites, only putative targets with predicted energy values less than or equal to − 15 kcal/mol were considered (Zhang et al. 2017); significance was defined at *P *< 0.05.

### Microarray data accession number

The gene expression data have been deposited in the Gene Expression Omnibus (GEO) database under accession number GSE124807.

## Results

### Growth properties of *E. coli* Trans10-*nfiS*

**Fig. 1:**
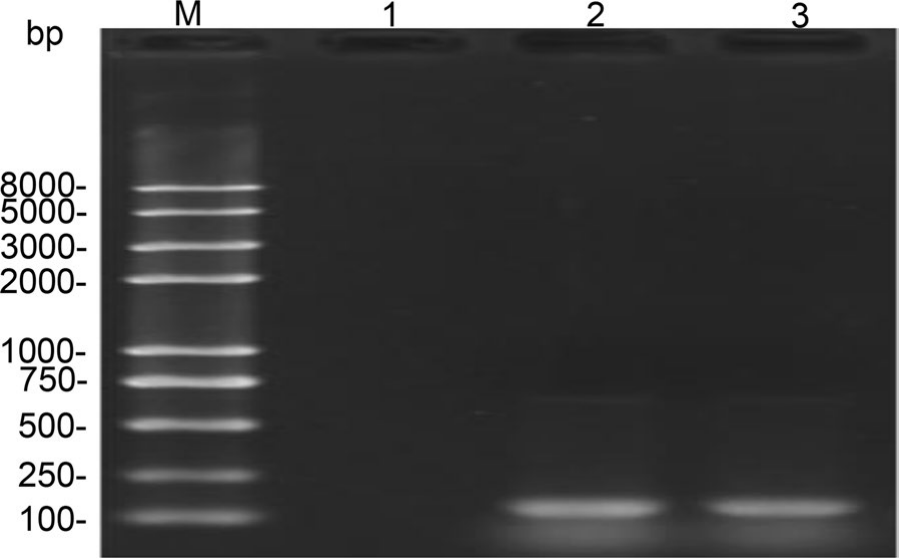
Confirmation of *nfiS* by RT-PCR. M: DNA ladder, 1: negative control (Trans 10); 2–3: positive clones (Trans10-*nfiS*)

We validated the introduction and expression of NfiS using RT-PCR to confirm the results of the triparental conjugation experiments. mRNA from Trans10-*nfiS* yielded a PCR product of 143 bp, which is consistent with the expected RT-PCR product size amplified with specific primer sets *nfiS*-F and *nfiS*-R. In contrast, no significant specific bands were observed when mRNA from Trans10 was used as a template during PCR amplification (Fig. 1). These data showed that *P. stutzeri* species-specific *nfiS* can be transcribed to yield noncoding RNA NfiS.

**Fig. 2:**
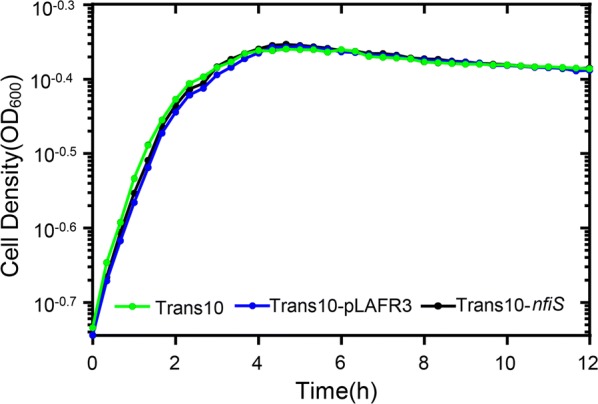
Semilogarithmic growth curve of WT Trans10 and Trans10-*nfiS* growth

To explore the effect of NfiS expression on the growth of *E. coli* Trans10, the growth performance of Trans10-*nfiS* was compared to that of Trans10. To eliminate interference from the mobilizable vector pLAFR3, a recombinant strain, Trans10-pLAFR3, harboring the empty pLAFR3 vector, was constructed and used as a negative control. As the semilogarithmic curves show, In LB medium, Trans10-*nfiS* exhibited a similar growth pattern to that of Trans10 (Fig. 2). It was concluded that introduction of the *nfiS* gene did not have any effect on the observable growth defect of *E. coli* Trans10 in LB medium.

### NfiS-expressing *E. coli* exhibits distinct oxidative and osmotic stress tolerance

**Fig. 3:**
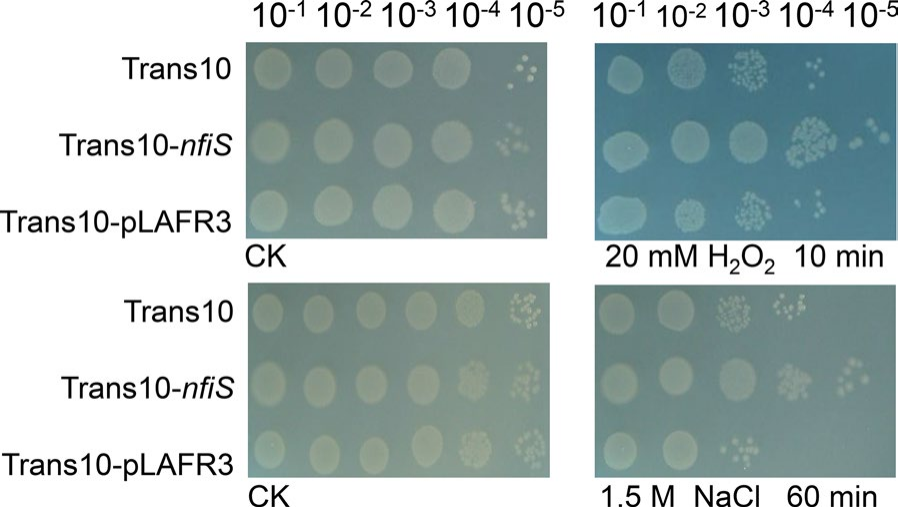
Growth under oxidative or osmotic stress. Serial tenfold dilutions of OD-standardized WT Trans10, Trans10-pLAFR3, and Trans10-*nfiS* were spotted on LB plates after exposure to 20 mM H_2_O_2_ or 1.5 M NaCl. CK, untreated culture control

Bacterial ncRNAs are involved in diverse stress responses. Among these ncRNAs, the *P. stutzeri* strain-derived RNA NfiS has been reported to be involved in the control of nitrogen fixation and the response to oxidative and osmotic stress. In the presence of 20 mM H_2_O_2_ or 1.5 M NaCl, heterologous expression of NfiS in *E. coli* led to enhanced resistance, whereas the Trans10 strain exhibited high sensitivity (Fig. 3).

To determine whether NfiS improved the ability of Trans10 to resist multiple adverse environments as a global regulator, the cell survival rate was estimated by testing serial dilutions from 10^−1^ to 10^−5^ under heat shock (53 °C), alkaline conditions (pH 10), acidic conditions (pH 2.0) and 0.3 M sorbitol treatment. The ability to resist different stress factors is reflected by the number of colonies on the plates. In these experiments, we did not observe any increase in the number of colonies of Trans10-*nfiS* relative to Trans10, indicating that NfiS was not involved in the tested stress regulation pathways. Based on these studies, NfiS likely controls the response to certain types of stress, especially oxidative stress response-related genes, via unknown mechanisms.

### Analysis of the *E. coli* Trans10-*nfiS* transcriptome under H_2_O_2_ shock conditions

**Fig. 4:**
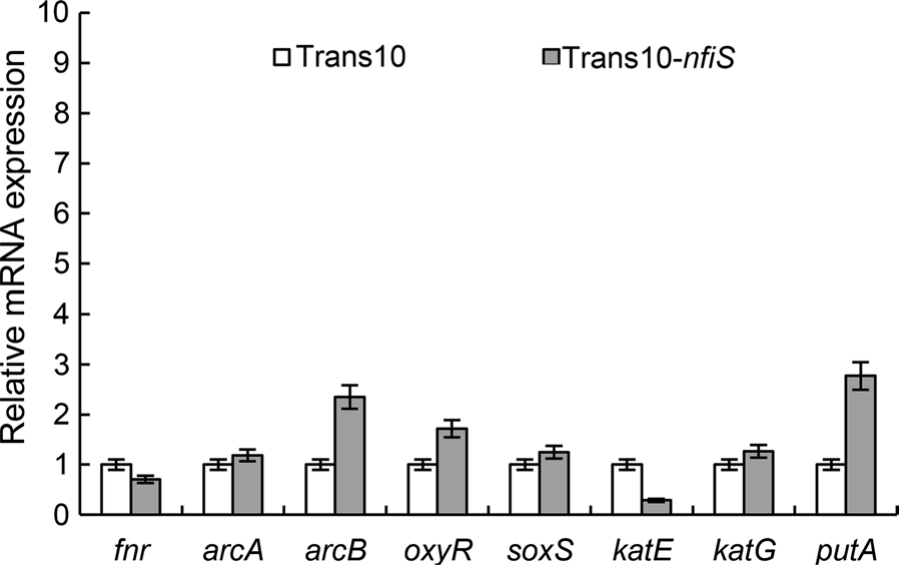
Effect of *nfiS* expression on the expression of oxidative genes in *E. coli*. Relative levels of transcripts are presented as the mean values ± SDs, calculated from three sets of independent experiments, and normalized to the levels in the control strain

The cell survival rate of Trans10-*nfiS* cells under H_2_O_2_ shock treatment was more distinguishable from that under osmotic stress, implying that NfiS may have a more essential role in regulating oxidative stress-related pathways. To clarify the potential mechanism of NfiS ncRNA in oxidative resistance, we performed DNA microarray analysis with both the recombinant Trans10-*nfiS* and WT *E. coli* Trans10 under identical H_2_O_2_ shock treatment conditions (see “Materials and methods” for details). The expression levels of several genes (two downregulated genes, *fnr*, *katE* and six upregulated genes, *soxS*, *oxyR*, *arcA*, *arcB*, *katG* and *putA*) observed in the microarray analyses were validated by using quantitative real-time PCR. Overall, these results were consistent with those obtained by transcriptome sequencing (Fig. 4).

**Fig. 5:**
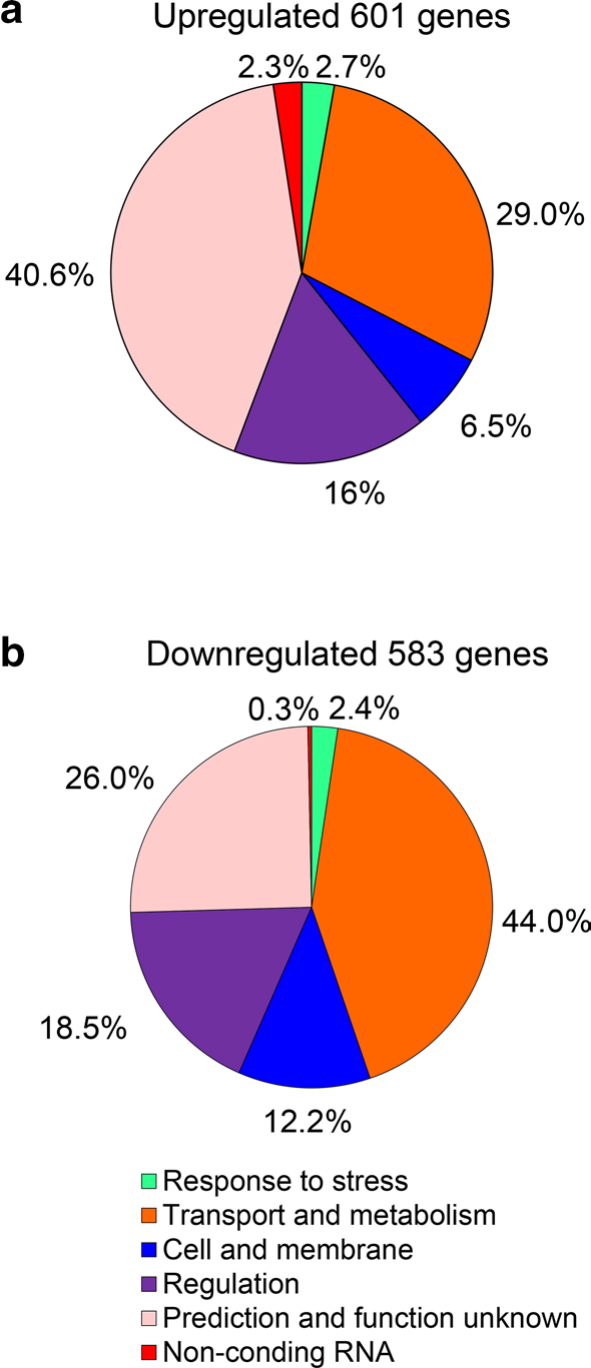
Overview of the expression profile of the recombinant nitrogen-fixing *E. coli* Trans10-*nfiS*. **a** Functional categories of the nitrogen fixation-induced genes (P ≤ 0.05) in Trans10-*nfiS*. **b** functional categories of the core subset of downregulated genes under 20 mM H_2_O_2_ treatment conditions

Our data showed that a total of 1184 genes were significantly altered (expression was changed by at least twofold) in Trans10-*nfiS* relative to Trans10, including 601 upregulated genes and 583 downregulated genes. These altered genes were further classified according to the COG functional classification system and were mainly divided into five functional categories (Fig. 5): stress response, regulation, metabolism related, transport or membrane protein and unknown function. Here, the oxidative stress response pathway is discussed in detail.

ROS overdose can cause a multitude of interrelated biochemical reactions in cells, including lipid peroxidation, covalent modification and oxidation of proteins and DNA lesions such as base damage, ultimately resulting in cell death (Blokhina et al. 2003). For *E. coli*, RpoS, OxyR, SoxSR and Fur were identified as the most crucial regulons for sensing ROS levels within the cell and subsequently controlling the expression of many specific genes or regulating target protein activities to protect organisms from the cytotoxic effects of oxidants. The differentially expressed RpoS OxyR, SoxSR and Fur regulons in two *E. coli* strains (Trans10 and Trans10-*nfiS*), both under H_2_O_2_ treatment, are listed in Additional file 1: Tables S2–S5, respectively.

For the 76 differentially expressed genes found in the RpoS regulon, the DNA repair exonuclease III gene *xthA*, which participates in oxidative-stress-induced DNA damage repair, was upregulated 7.75-fold in Trans10-*nfiS* relative to Trans10. The DNA mismatch repair protein gene *mutS* was upregulated by 3.38-fold. This may indicate that NfiS enhanced the DNA damage repair system to protect the cell from oxidative damage. The 39 genes regulated by OxyR can be divided into two categories: (1) genes involved in the direct elimination of H_2_O_2_ (*katG*, *ahpC*, *ahpF*, *yhjA* and *hemF*) and (2) genes involved in the regulation of redox potential (*grx*, *gor*, *trxB*, *trxC*, *fhuF* and *dsbG*). Microarray data showed that the expression of *katG* (hydroperoxidase I), *ahpC*, *ahpF* (alkyl hydroperoxide reductase component), *yhjA* (cytochrome c peroxidase) and *hemF* (coproporphyrinogen III oxidase) remained almost unchanged, while the glutathione reductase gene *gor* and thioredoxin reductase gene *trxB* were upregulated 2.97-fold and 7.28-fold, respectively (Additional file 1: Table S3). Thus, it is possible that NfiS, rather than direct clearance of H_2_O_2_, improved *E. coli* oxidation resistance via accumulation of glutathione reductase/thioredoxin reductase (Sengupta and Holmgren 2014), which is responsible for the oxidative-state activation of OxyR. A previous study revealed that numerous genes involved in oxidative stress resistance were SoxRS-dependent, including *sodA* (MnSOD), *nfo* (DNA repair endonuclease IV), *zwf* (glucose-6-phosphate dehydrogenase, G6PD), *acnA* (aconitate hydratase), *fumC* (fumarase), *acrAB* (efflux pump), *fpr* (flavodoxin/ferredoxin-NADP(+) reductase), *fur* (Fe uptake and regulator), *fldAB* (flavodoxin), and *micF* (Chiang and Schellhorn 2012). Among these genes, *gcd* (glucose dehydrogenase) was upregulated 3.19-fold, *acrA* was upregulated 3.12-fold and *acrB* was upregulated slightly as well as in Trans10-*nfiS* (Additional file 1: Table S4). No significant changes in *fpr, fur, nfo, zwf, sodA* and *fldAB* gene expression were detected, indicating that the enhancement of stress resistance was unrelated to these genes. *fur* has been reported to be activated by OxyR and SoxR, which are known regulators of oxidative stress (Zheng et al. 1999). Given that iron-sulfur cluster synthesis is susceptible to superoxide anions induced by H_2_O_2_ stress, the expression of the Fur regulon genes *iscAUSRX* (Fe–S cluster), *fdx* (reduced ferredoxin), *hscAB* (cochaperone for Fe–S cluster biosynthesis), *pepB* (aminopeptidase B)*, sseB* (enhanced serine sensitivity protein) and *sufESDCBA* (Fe–S cluster assembly protein) were downregulated by different degrees. Of these genes, *fdx and iscX* were downregulated 2.22-fold and 2.26-fold, respectively. The decrease was further enhanced for the *suf* operon, and the genes *sufE, sufS, sufD and sufC* were downregulated 11.73-fold, 7.24-fold, 3.01-fold and 2.46-fold, respectively (Additional file 1: Table S5). Based on these studies, NfiS controls Fe–S cluster synthesis at the transcriptional level via regulation of both the OxyR/SoxRS regulatory cascade and certain stress response-related genes.

The expression of the cysteine synthesis-associated gene cluster (*cys*) and associated genes generally decreased (Additional file 1: Table S6), since cysteine residues and Fe–S clusters are sensitive to the ROS generated under H_2_O_2_ treatment. Superoxide anions disrupt iron-sulfur clusters, leading to the release of Fe^2+^, which reacts with H_2_O_2_ to form hydroxyl free radicals. The hydroxyl free radical is a type of strong oxidant that directly damages cysteine residues. H_2_O_2_ can also directly oxidize the cysteine residues of proteins. Superoxide anions and the denitrification intermediate nitric oxide anions form oxygen nitrite, which diffuses into cells and damages cysteine residues, so cysteine is often damaged as a direct target of ROS produced under oxidative stress.

Ribonucleotide reductases are widely present in prokaryotes. The biosynthesis of NTPs, the basic units for DNA replication, is catalyzed by the ribonucleotide reductase encoded by the *nrd* gene cluster (Torrents 2014). Via specific binding to four types of dNTPs, the active site of ribonucleotide reductase provides the substrate that is indispensable for the *E. coli* DNA damage repair system. Compared with Trans10 under oxidative stress conditions, Trans10-*nfiS nrdHIEF* mRNA expression increased 10.26-fold, 6.14-fold, 1.78-fold and 9.03-fold, respectively (Additional file 1: Table S7), indicating that the DNA damage repair ability of the strain was enhanced by the introduction of NfiS.

Biotin, also known as vitamin B7, is a necessary vitamin for growth. The synthesis of biotin in *E. coli* starts from heptanoic acid and is mainly completed by enzymes in the biosynthetic pathway (Eisenberg and Star 1968). The process is catalyzed by heptanoyl coenzyme A synthase, the 7-ketol-8 amino-pelargonic acid synthase BioF, the 7,8-diaminonucanoic acid synthase BioA, the desulfurization synthase BioD and the biotin synthase BioB. Under oxidative stress, biotin synthesis pathway genes in the NfiS expression strain were inhibited. For example, the expression levels of *bioF, bioD* and *bioB* were downregulated 3-fold, 3.17-fold and 1.8-fold, respectively (Additional file 1: Table S8).

Similarly, most of the acid resistance genes were downregulated more than twofold (Additional file 1: Table S9). There are four types of metabolites involved in the acid resistance pathways: glucose, arginine, glutamic acid and lysine (Richard and Foster 2004; Yoo et al. 2013). For the latter three acid resistance systems, in the presence of each of the amino acids, the decarboxylase can deacidify the amino acid and continuously consume intracellular protons, preventing extracellular protons from flowing into the cell and reducing the intracellular pH value to alleviate the damage caused by the low extracellular pH. However, compared with Trans10, the expression of glutamic acid-dependent genes was significantly inhibited in Trans10-*nfiS*. The glutamic acid decarboxylases *gadA* and *gadB* were downregulated 11.5-fold and 11.6-fold, respectively. The glutamate and γ-aminobutyric acid reverse transporter gene *gadC* was downregulated 6.96-fold. We speculate that Trans10-*nfiS* is induced to express antioxidant genes by consuming ATP and NADPH, which leads to depletion of the substrates required for resistance to acid stress and for energy, eventually suppressing the expression of acid resistance genes.

Hfq is an important molecular chaperone reported to be involved in sRNA activity and function (Vytvytska et al. 2000). In this study, we did not find a significant change of Hfq in Trans10 due to the introduction of *nfiS*.

### In silico prediction of genes as targets of NfiS in *E. coli*

To investigate potential regulation by NfiS, we next performed an in silico analysis using the IntaRNA program. The output table summarizes the best 100 predicted interactions (Additional file 1: Table S10), but only the top 25 target genes (P-value lower than 0.5) were selected for further discussion (Table 2). A region within the leader of *oweS* (b2358, also known as *yfdO*) mRNA and a region within the coding sequence (CDS) of *xanP* (b3654, also known as *yicE*) mRNA were confirmed as the high-scoring interaction sites for NfiS. Interestingly, the predicted gene *oweS* (encoding prophage CPS-53 protein YfdO) has been reported to enhance resistance to oxidative stress (Wang et al. 2010). Moreover, prophages containing YfdO provide multiple benefits (withstanding osmotic, oxidative and acid stresses, increasing growth, and influencing biofilm formation) to the host for survival under adverse environmental conditions. The YicE protein is a member of the NCS2 family of nucleobase transporters. YicE was shown to be present in the plasma membrane of *E. coli* and function as specific, high-affinity transporter for xanthine in a proton motive force-dependent manner, which is an essential process that generates functional substrates needed for the repair of double-strand breaks in *E. coli*. Thus, our computational analysis suggests that NfiS may directly regulate *oweS* and *xanP* mRNA expression in *E. coli* Trans10.

**Table 2:** NfiS target prediction

Putative target	Gene	Fold change	Annotation	P-value	Energy
b2358	*oweS*	− 1.11	Pseudogene CPS-53 (KpLE1) prophage bacteriophage replication protein O family phage or prophage related	6.96E−05	− 34.32
b3654	*xanP*	1.03	Xanthine permease	6.96E−05	− 34.31
b2536	*hcaT*	− 1.02	Putative 3-phenylpropionic transporter	5.03E−04	− 30.89
b3603	*lldP*	2.13	L-lactate permease	6.31E−04	− 30.47
b2576	*srmB*	1.49	ATP-dependent RNA helicase	6.91E−04	− 30.29
b2611	*ypjD*	3.41	Cytochrome c assembly protein family inner membrane protein	7.96E−04	− 30.03
b3783	*rho*	− 4.69	Transcription termination factor	1.18E−03	− 29.28
b3096	*mzrA*	− 2.50	Modulator of EnvZ/OmpR regulon	1.22E−03	− 29.21
b1061	*dinI*	− 1.93	DNA damage-inducible protein I	1.23E−03	− 29.20
b3060	*ttdR*	− 1.06	Transcriptional activator of *ttdABT*	1.28E−03	− 29.11
b1125	*potB*	1.05	Spermidine/putrescine ABC transporter permease	1.53E−03	− 28.76
b0920	*elyC*	2.64	Envelope biogenesis factor DUF218 superfamily protein	1.56E−03	− 28.73
b2135	*yohC*	− 4.70	Yip1 family inner membrane protein	2.18E−03	− 28.06
b2475	*ypfJ*	− 1.26	Putative neutral zinc metallopeptidase	2.24E−03	− 28.00
b0338	*cynR*	2.07	Transcriptional activator of *cyn* operon autorepressor	2.32E−03	− 27.93
b3592	*yibF*	− 1.38	Glutathione S-transferase homolog	2.58E−03	− 27.72
b3234	*degQ*	1.06	Serine endoprotease periplasmic	2.94E−03	− 27.45
b3946	*fsaB*	1.74	Fructose-6-phosphate aldolase 2	3.38E−03	− 27.16
b3413	*gntX*	1.73	DNA catabolic protein	3.60E−03	− 27.03
b0269	*yagF*	− 17.39	CP4-6 prophage dehydratase family protein	3.89E−03	− 26.86
b0875	*aqpZ*	− 3.05	Aquaporin Z	4.12E−03	− 26.74
b3115	*tdcD*	1.58	Propionate kinase/acetate kinase C anaerobic	4.52E−03	− 26.55
b0089	*ftsW*	− 1.48	Putative lipid II flippase integral membrane protein FtsZ ring stabilizer	4.60E−03	− 26.51
b1859	*znuB*	1.00	Zinc ABC transporter permease	4.87E−03	− 26.39
b2832	*ygdQ*	1.84	UPF0053 family inner membrane protein	4.89E−03	− 26.38

The impact of NfiS on *oweS* and *xanP* gene expression was less profound compared to the impact on *ypjD* (encoding cytochrome c assembly family protein), *rho* (encoding transcription termination factor Rho), *mzrA* (encoding modulator of EnvZ/OmpR regulon), *elyC* (encoding envelope biogenesis factor), *yohC* (putative inner membrane protein), *cynR* (encoding DNA-binding transcriptional dual regulator CynR), *yagF* (encoding D-xylonate dehydratase) and *aqpZ* (water channel AqpZ) estimated by transcriptome profiling, although there had been no previous report that these differentially expressed genes are involved in the antioxidant regulatory pathway. At present, whether they can bind to NfiS under oxidative stress conditions remains uncertain.

## Discussion

It has been suggested that the regulation of ncRNA diversity provides an opportunity for the generation of novel functional strains by genetic engineering or synthetic biological methods. It has been reported that, NfiS, coordinates oxidative stress response and nitrogen fixation via base pairing with *katB* mRNA and *nifK* mRNA (Zhang et al. 2019). This work examines NfiS, an ncRNA specific to *P. stutzeri*, which plays a similar role in *E. coli* as an antioxidant. Heterologous expression of NfiS in Trans10 cells enhanced the oxidative stress resistance process, which is consistent with the role of NfiS in *P. stutzeri.* In addition, examination of differential gene expression in recombinant *E. coli* identified an array of genes associated with ROS clearance. It is proposed that the primary function of NfiS in *P. stutzeri* is associated with the stress response and that NfiS likely contains another nucleotide sequence that pairs with other, as-yet-unidentified target genes.

To the best of our knowledge, *E. coli* has several major regulators that are up-regulated during oxidative stress, including OxyR, SoxRS, RpoS and Fur. OxyR negatively regulates the expression of its encoding gene (*oxyR*) and positively regulates an adjacent small RNA gene (*oxyS*). OxyR controls a regulon of almost 40 genes, which protect the cell from the toxic effects of H_2_O_2_. The *soxR* and *soxS* are adjacent and divergently transcribed in *E. coli* (Wu and Weiss 1991). Proteins encoded by *soxR* and *soxS* constitute a two-stage regulatory system in which SoxR, when activated, induces the expression of *soxS*, which in turn regulates several genes that are important for the oxidative stress response. OxyR and SoxR are activated by conformational changes due to oxidization, whereas RpoS leads to increased recruitment of RNA polymerase to RpoS-dependent promoters. To some extent, Iron homeostasis and the oxidative stress response are linked. OxyR and SoxRS are capable of induce Fur expression, via the binding of OxyR to the *fur* promoter and the binding of SoxS to the upstream *fldA* (encoding flavodoxin) promoter (Varghese et al. 2007). Fur activity can be diminished in the H_2_O_2_^−^ containing environments and OxyR-mediated induction of Fur was found to help alleviate the loss of Fur activity (Pohl et al. 2003). However, in *P. stutzeri* A1501, genes involved in ROS resistance and protection from oxidative stress remain to be studied.

Instead of obtaining all genes involved in oxidative stress, the purpose of the transcriptome assay design was to excavate possible targets of the ncRNA. By using genome-wide microarray analysis, changes in the expression levels of genes involved in oxidative resistance were detected under 20 mM H_2_O_2_ treatment. According to the four major regulators activated during oxidative stress, these genes can be divided into OxyR-, SoxRS-, RpoS- and Fur-dependent genes. Among these genes, the *gor, trx, agn43* and *uxuA* genes regulated by OxyR (Wallecha et al. 2014) were upregulated more than twofold. Additionally, SoxR-dependent genes, such as *yggX*, *gcd* and *acrAB*, and the RpoS-dependent genes *xthA* and *mutS* were upregulated. Homeostatic control of free intracellular iron levels is important for minimization of oxidative stress. Fur binds to DNA at a Fur box to repress iron acquisition genes, which is consistent with a role for Fur in oxidative stress resistance. The expression levels of the *isc* operon and *suf* operon (Fe–S cluster formation) were decreased significantly. It is hypothesized that NfiS controls these target genes to participate in the oxidative resistance of *E. coli*. In addition, NfiS affects the synthesis of cysteine and biotin in the cell, promotes the activity of ribonucleotide reductase, inhibits the synthesis of iron-sulfur clusters and affects the expression of the glutamate-dependent acid tolerance system, and these biological pathways or processes can also play important roles in enhancing the antioxidant tolerance of *E. coli*.

We noticed that *rpoS*, *oxyR*, *soxSR* and *fur* were not among the differentially expressed genes, which does not rule out important roles for those genes in oxidative resistance since the transcriptome analyses were designed to explore pathways influenced by NfiS. It is possible that many vital regulators involved in oxidative stress tolerance were expressed highly within both Trans10 and Trans10-*nfiS*, but this was not reflected by the transcriptome results. In summary, the *nfiS* gene cloned from *P. stutzeri* A1501 has been shown to protect *E. coli* from ROS. Many antioxidant genes in *E. coli* could be identified using differential transcriptomic analysis, and a potential mechanism of action for NfiS was identified. Bacterial small RNAs (sRNAs) have been implicated in various aspects of post-transcriptional gene regulation without modification of chromosomal sequences. Various strategies for systems metabolic engineering can be used for further strain improvement followed the superior platform strain and effective sRNA target genes have been identified. The work presented here enables the rapid development of high-performance microbial strains.

## Supplementary information

**Additional file 1.** Additional tables.

## Data Availability

The datasets supporting the conclusions of this article are included within the article.

## Abbreviations

ncRNA: noncoding RNA
A1501: *Pseudomonas stutzeri* A1501
WT: wild type
Tc: tetracycline
OD: optical density
RT-PCR: reverse transcription PCR
qRT-PCR: quantitative real-time PCR
bp: base pair
SD: standard deviation

## References

[CR1] Blokhina O, Virolainen E, Fagerstedt KV (2003). Antioxidants, oxidative damage and oxygen deprivation stress: a review. Ann Bot.

[CR2] Busch A, Richter AS, Backofen R (2008). IntaRNA: efficient prediction of bacterial sRNA targets incorporating target site accessibility and seed regions. Bioinformatics.

[CR3] Chiang SM, Schellhorn HE (2012). Regulators of oxidative stress response genes in *Escherichia coli* and their functional conservation in bacteria. Arch Biochem Biophys.

[CR4] Desnoues N, Lin M, Guo X, Ma L, Carreno-Lopez R, Elmerich C (2003). Nitrogen fixation genetics and regulation in a *Pseudomonas stutzeri* strain associated with rice. Microbiology.

[CR5] Eisenberg MA, Star C (1968). Synthesis of 7-oxo-8-aminopelargonic acid, a biotin vitamer, in cell-free extracts of *Escherichia coli* biotin auxotrophs. J Bacteriol.

[CR6] Gonzalez N, Heeb S, Valverde C, Kay E, Reimmann C, Junier T, Haas D (2008). Genome-wide search reveals a novel GacA-regulated small RNA in *Pseudomonas* species. BMC Genomics.

[CR7] González-Flecha B, Demple B (1999). Role for the *oxyS* gene in regulation of intracellular hydrogen peroxide in *Escherichia coli*. J Bacteriol.

[CR8] Imlay JA (2008). Cellular defenses against superoxide and hydrogen peroxide. Annu Rev Biochem.

[CR9] Lenz DH, Miller MB, Zhu J, Kulkarni RV, Bassler BL (2005). CsrA and three redundant small RNAs regulate quorum sensing in *Vibrio cholerae*. Mol Microbiol.

[CR10] Liang JC, Bloom RJ, Smolke CD (2011). Engineering biological systems with synthetic RNA molecules. Mol Cell.

[CR11] McHugh JP, Rodriguez-Quinones F, Abdul-Tehrani H, Svistunenko DA, Poole RK, Cooper CE, Andrews SC (2003). Global iron-dependent gene regulation in *Escherichia coli*. A new mechanism for iron homeostasis. J Biol Chem.

[CR12] Na D, Yoo SM, Chung H, Park H, Park JH, Lee SY (2013). Metabolic engineering of *Escherichia coli* using synthetic small regulatory RNAs. Nat Biotechnol.

[CR13] Nakamura T, Naito K, Yokota N, Sugita C, Sugita M (2007). A cyanobacterial non-coding RNA, Yfr1, is required for growth under multiple stress conditions. Plant Cell Physiol.

[CR14] Negrete A, Shiloach J (2017). Improving *E. coli* growth performance by manipulating small RNA expression. Microb Cell Fact.

[CR15] Park SH, Butcher BG, Anderson Z, Pellegrini N, Bao Z, D’Amico K, Filiatrault MJ (2013). Analysis of the small RNA P16/RgsA in the plant pathogen *Pseudomonas syringae* pv. tomato strain DC3000. Microbiology.

[CR16] Pohl E, Haller JC, Mijovilovich A, Meyer-Klaucke W, Garman E, Vasil ML (2003). Architecture of a protein central to iron homeostasis: crystal structure and spectroscopic analysis of the ferric uptake regulator. Mol Microbiol.

[CR17] Richard H, Foster JW (2004). *Escherichia coli* glutamate- and arginine-dependent acid resistance systems increase internal pH and reverse transmembrane potential. J Bacteriol.

[CR18] Romby P, Vandenesch F, Wagner EG (2006). The role of RNAs in the regulation of virulence-gene expression. Curr Opin Microbiol.

[CR19] Sengupta R, Holmgren A (2014). Thioredoxin and glutaredoxin-mediated redox regulation of ribonucleotide reductase World. J Biol Chem.

[CR20] Skippington E, Ragan MA (2012). Evolutionary dynamics of small RNAs in 27 *Escherichia coli* and *Shigella* genomes. Genome Biol Evol.

[CR21] Staskawicz B, Dahlbeck D, Keen N, Napoli C (1987). Molecular characterization of cloned avirulence genes from race 0 and race 1 of *Pseudomonas syringae* pv. *glycinea*. J Bacteriol.

[CR22] Torrents E (2014). Ribonucleotide reductases: essential enzymes for bacterial life. Front Cell Infect Microbiol.

[CR23] Trotter EW, Grant CM (2003). Non-reciprocal regulation of the redox state of the glutathione–glutaredoxin and thioredoxin systems. EMBO Rep.

[CR24] Varghese S, Wu A, Park S, Imlay KR, Imlay JA (2007). Submicromolar hydrogen peroxide disrupts the ability of Fur protein to control free-iron levels in *Escherichia coli*. Mol Microbiol.

[CR25] Vytvytska O, Moll I, Kaberdin VR, von Gabain A, Bläsi U (2000). Hfq (HF1) stimulates *ompA* mRNA decay by interfering with ribosome binding. Genes Dev.

[CR26] Wallecha A, Oreh H, van der Woude MW, deHaseth PL (2014). Control of gene expression at a bacterial leader RNA, the *agn43* gene encoding outer membrane protein Ag43 of *Escherichia coli*. J Bacteriol.

[CR27] Wang X, Kim Y, Ma Q, Hong SH, Pokusaeva K, Sturino JM, Wood TK (2010). Cryptic prophages help bacteria cope with adverse environments. Nat Commun.

[CR28] Wassarman KM, Zhang AX, Storz G (1999). Small RNAs in *Escherichia coli*. Trends Microbiol.

[CR29] Wu J, Weiss B (1991). Two divergently transcribed genes, soxR and soxS, control a superoxide response regulon of *Escherichia coli*. J Bacteriol.

[CR30] Yan Y, Yang J, Dou Y, Chen M, Ping S, Peng J, Lu W, Zhang W, Yao Z, Li H, Liu W, He S, Geng L, Zhang X, Yang F, Yu H, Zhan Y, Li D, Lin Z, Wang Y, Elimerich C, Lin M, Jin Q (2008). Nitrogen fixation island and rhizosphere competence traits in the genome of root-associated *Pseudomonas stutzeri* A1501. Proc Natl Acad Sci USA.

[CR31] Yoo SM, Na D, Lee SY (2013). Design and use of synthetic regulatory small RNAs to control gene expression in *Escherichia coli*. Nat Protoc.

[CR32] Yuan X, Khokhani D, Wu X, Yang F, Biener G, Koestler BJ, Raicu V, He C, Waters CM, Sundin GW, Tian F, Yang CH (2015). Cross-talk between a regulatory small RNA, cyclic-di-GMP signalling and flagellar regulator FlhDC for virulence and bacterial behaviours. Environ Microbiol.

[CR33] Zhan Y, Yan Y, Deng Z, Chen M, Lu W, Lu C, Shang L, Yang Z, Zhang W, Wang W, Li Y, Ke Q, Lu J, Xu Y, Zhang L, Xie Z, Cheng Q, Elmerich C, Lin M (2016). The novel regulatory ncRNA, NfiS, optimizes nitrogen fixation via base pairing with the nitrogenase gene *nifK* mRNA in *Pseudomonas stutzeri* A1501. Proc Natl Acad Sci USA.

[CR34] Zhang Y, Yan D, Xia L, Zhao X, Osei-Adjei G, Xu S, Sheng X, Huang X (2017). The *malS*-5′UTR regulates *hisG*, a key gene in the histidine biosynthetic pathway in *Salmonella enterica* serovar Typhi. Can J Microbiol.

[CR35] Zhang H, Zhan Y, Yan Y, Liu Y, Hu G, Wang S, Yang H, Qiu X, Liu Y, Li J, Lu W, Elmerich C, Lin M (2019). The *Pseudomonas stutzeri*-specific regulatory ncRNA, NfiS, targets the *katB* mRNA encoding a catalase essential for optimal oxidative resistance and nitrogenase activity. J Bacteriol.

[CR36] Zheng M, Doan B, Schneider TD, Storz G (1999). OxyR and SoxRS regulation of *fur*. J Bacteriol.

