# Supplementary material for: NfiS, a species-specific regulatory noncoding RNA of Pseudomonas stutzeri, enhances oxidative stress tolerance in Escherichia coli
Source: AMB Express. 2019 Sep 25;9:156. doi: 10.1186/s13568-019-0881-7 (PMC6761216; doi:10.1186/s13568-019-0881-7)
Supplement: Supplementary file 1 — Additional file 1. Additional tables. [file 13568_2019_881_MOESM1_ESM.docx]

**Additional Tables:**

Table S1. Primers used in qRT-PCR experiment

| Primer | Sequence (5’ to 3’) |
| --- | --- |
| RT*fnr*-F | ACTGAGCAAGGCGACGAG |
| RT*fnr*-R | CGACGGGACAGGTTGTAGAT |
| RT*arcA*-F | CGAAGCGGAAGGCTATGA |
| RT*arcA*-R | GGGTTGAACGGTTTGGTG |
| RT*arcB*-F | GCGCAGTATTATGTTGACC |
| RT*arcB*-R | CCACCGATAGAAAGTAGACC |
| RT*oxyR*-F | GGGAATGCTGCTGGTGGA |
| RT*oxyR*-R | AACTGGTGGGTCTGTGCTT |
| RT*soxS*-F | AATCAGGCTATTCAAAGTGG |
| RT*soxS*-R | GATAATCGCTGGGAGTGC |
| RT*katE*-F | AGTGCCCACGATACTTTCT |
| RT*katE*-R | CAATGGCTTCCCACAACT |
| RT*katG*-F | CGGTTTCCGTAACTATCGC |
| RT*katG*-R | ATTCGTCGGTCGCTTTCC |
| RT*putA*-F | CCCTGCGGAACCGAAAGA |
| RT*putA*-R | CGGCAATGGCGTTACTGA |

Table S2. Expression of RpoS regulon genes in the Trans10-*nfiS* strain

| Blattner number | Gene name | Functional description | Log_2_ ratio |
| --- | --- | --- | --- |
| Upregulated | | | |
| b0243 | *proA* | gamma-glutamyl phosphate reductase | 1.22 |
| b0329 | *yahO* | predicted protein | 7.24 |
| b1073 | *flgB* | flagellar component of cell-proximal portion of basal-body rod | 1.21 |
| b1076 | *flgE* | flagellar hook protein | 1.09 |
| b1078 | *flgG* | flagellar component of cell-distal portion of basal-body rod | 1.19 |
| b1256 | *ompW* | outer membrane protein W | 1.43 |
| b1339 | *abgR* | predicted DNA-binding transcriptional regulator of *abgABT* operon | 1.22 |
| b1749 | *xthA* | exonuclease III | 2.92 |
| b2733 | *mutS* | methyl-directed mismatch repair protein | 1.76 |
| b2784 | *relA* | (p)ppGpp synthetase I/GTP pyrophosphokinase | 1.34 |
| b3500 | *gor* | glutathione oxidoreductase | 1.57 |
| b4314 | *fimA* | major type 1 subunit fimbrin (pilin) | 5.50 |
| Downregulated | | | |
| b0435 | *bolA* | stationary-phase morphogene, transcriptional repressor for *mreB*; regulator for *dacA*, *dacC*, and *ampC* | -1.09 |
| b0485 | *ybaS* | glutaminase | -2.63 |
| b0486 | *ybaT* | predicted transporter | -3.04 |
| b0453 | *ybaY* | outer membrane lipoprotein | -1.56 |
| b0553 | *nmpC* | *nmpC*: pseudo | -5.82 |
| b0753 | *ybgS* | conserved protein | -2.26 |
| b0897 | *ycaC* | predicted hydrolase, isochorismatase family | -2.63 |
| b1003 | *yccJ* | predicted protein | -2.28 |
| b1051 | *msyB* | multicopy suppressor of *secY* and *secA* | -2.43 |
| b1100 | *ycfH* | predicted DNase | -2.10 |
| b1167 | *ymgC* | predicted protein | -1.12 |
| b1188 | *ycgB* | conserved protein | -2.76 |
| b1195 | *ymgE* | predicted inner membrane protein | -1.89 |
| b1197 | *treA* | periplasmic trehalase | -1.01 |
| b1334 | *fnr* | DNA-binding transcriptional dual regulator, global regulator of anaerobic growth | -1.28 |
| b1337 | *abgB* | p-aminobenzoyl-glutamate hydrolase, B subunit | -1.13 |
| b1344 | *ttcA* | tRNA 2-thiocytidine biosynthesis protein | -1.15 |
| b1345 | *intR* | Rac prophage; integrase | -1.01 |
| b1352 | *kilR* | Rac prophage; inhibitor of *ftsZ*, killing protein | -1.44 |
| b1355 | *ydaG* | Rac prophage; predicted protein | -1.97 |
| b1482 | *osmC* | Lipoyl-dependent Cys-based peroxidase, hydroperoxide resistance; salt-shock inducible membrane protein; peroxiredoxin | -1.11 |
| b1492 | *gadC* | predicted glutamate: gamma-aminobutyric acid antiporter | -2.80 |
| b1493 | *gadB* | glutamate decarboxylase B, PLP-dependent | -3.54 |
| b1536 | *ydeI* | conserved protein | -1.50 |
| b1646 | *sodC* | superoxide dismutase, Cu, Zn | -1.93 |
| b1661 | *cfa* | cyclopropane fatty acyl phospholipid synthase (unsaturated-phospholipid methyltransferase) | -1.74 |
| b1678 | *ynhG* | murein L, D-transpeptidase | -2.43 |
| b1680 | *sufS* | cysteine desulfurase, stimulated by SufE; selenocysteine lyase, PLP-dependent | -2.86 |
| b1681 | *sufD* | FeS cluster assembly protein of SufBCD complex | -1.59 |
| b1724 | *ydiZ* | predicted protein | -1.27 |
| b1732 | *katE* | hydroperoxidase HPII(III) (catalase) | -1.28 |
| b1739 | *osmE* | DNA-binding transcriptional activator | -1.88 |
| b1783 | *yeaG* | protein kinase, function unknown; autokinase | -2.53 |
| b1896 | *otsA* | trehalose-6-phosphate synthase | -2.59 |
| b1897 | *otsB* | trehalose-6-phosphate phosphatase, biosynthetic | -3.29 |
| b1953 | *yodD* | predicted protein | -3.58 |
| b1957 | *yodC* | predicted protein | -1.28 |
| b1967 | *hchA* | Hsp31 molecular chaperone | -1.57 |
| b2080 | *yegP* | conserved protein, UPF0339 family | -3.48 |
| b2097 | *fbaB* | fructose-bisphosphate aldolase class I | -1.67 |
| b2135 | *yohC* | inner membrane protein, Yip1 family | -2.23 |
| b2266 | *elaB* | conserved protein | -2.79 |
| b2464 | *talA* | transaldolase A | -1.86 |
| b2465 | *tktB* | transketolase 2, thiamin-binding | -2.41 |
| b2665 | *ygaU* | predicted protein | -1.90 |
| b2742 | *nlpD* | activator of AmiC murein hydrolase activity, lipoprotein | -1.05 |
| b3097 | *yqjC* | conserved protein | -1.70 |
| b3099 | *yqjE* | inner membrane protein, DUF1469 family | -2.76 |
| b3239 | *yhcO* | predicted barnase inhibitor | -2.84 |
| b3336 | *bfr* | bacterioferritin, iron storage and detoxification protein | -1.92 |
| b3506 | *slp* | outer membrane lipoprotein | -2.24 |
| b3509 | *hdeB* | acid-resistance protein | -1.46 |
| b3510 | *hdeA* | stress response protein acid-resistance protein | -1.63 |
| b3511 | *hdeD* | acid-resistance membrane protein | -1.80 |
| b3512 | *gadE* | DNA-binding transcriptional activator | -1.44 |
| b3515 | *gadW* | transcriptional activator of *gadA* and *gadBC* in absence of GadX | -2.36 |
| b3516 | *gadX* | DNA-binding transcriptional dual regulator | -2.70 |
| b3517 | *gadA* | glutamate decarboxylase A, PLP-dependent | -3.53 |
| b3555 | *yiaG* | predicted transcriptional regulator, HTH_CROC1 family | -2.80 |
| b4111 | *proP* | proline/glycine betaine transporter | -1.80 |
| b4126 | *yjdI* | conserved protein | -1.68 |
| b4155 | *poxA* | regulatory gene for *poxB* | -1.09 |
| b4187 | *aidB* | isovaleryl CoA dehydrogenase | -1.76 |
| b4376 | *osmY* | periplasmic protein | -2.34 |

Table S3. Expression of OxyR regulon genes in the Trans10-*nfiS* strain

| Blattner number | Gene name | Functional description | Log_2_ ratio |
| --- | --- | --- | --- |
| Upregulated | | | |
| b0006 | *yaaA* | conserved protein, UPF0246 family | 1.75 |
| b0163 | *yaeH* | conserved protein, UPF0325 family | 0.33 |
| b0605 | *ahpF* | alkyl hydroperoxide reductase, C22 subunit | 0.30 |
| b0606 | *ahpC* | alkyl hydroperoxide reductase, F52a subunit, FAD/NAD(P)-binding | 0.22 |
| b0683 | *fur* | DNA-binding transcriptional dual regulator of siderophore biosynthesis and transport | 0.36 |
| b0812 | *dps* | Fe-binding and storage protein | 0.16 |
| b0872 | *hcr* | HCP oxidoreductase, NADH-dependent | 0.15 |
| b0881 | *yljA* | regulatory protein for ClpA substrate specificity | 0.49 |
| b0888 | *trxB* | thioredoxin reductase, FAD/NAD(P)-binding | 2.86 |
| b1426 | *ydcH* | predicted protein | 0.41 |
| b2000 | *flu* | CP4-44 prophage; antigen 43 (Ag43) phase-variable biofilm formation autotransporter | 1.46 |
| b2350 | *gtrA* | CPS-53 (KpLE1) prophage; bactoprenol-linked glucose translocase (flippase) | 0.55 |
| b2351 | *gtrB* | CPS-53 (KpLE1) prophage; bactoprenol glucosyl transferase | 0.30 |
| b2392 | *mntH* | manganese/divalent cation transporter | 0.78 |
| b2582 | *trxC* | thioredoxin 2 | 0.18 |
| b3500 | *gor* | glutathione oxidoreductase | 1.57 |
| b3518 | *yhjA* | predicted cytochrome C peroxidase | 0.81 |
| b3942 | *katG* | catalase/hydroperoxidase HPI(I) | 0.24 |
| b3961 | *oxyR* | DNA-binding transcriptional dual regulator | 0.67 |
| b4217 | *ytfK* | conserved protein, DUF1107 family | 0.55 |
| b4322 | *uxuA* | mannonate hydrolase | 5.80 |
| b4458 | *oxyS* | *oxyS*: misc_RNA ncRNA | 0.19 |
| Downregulated | | | |
| b0389 | *yaiA* | predicted protein | -3.50 |
| b0475 | *hemH* | ferrochelatase | -0.43 |
| b0604 | *dsbG* | thiol: disulfide interchange protein, periplasmic | -0.70 |
| b0848 | *ybjM* | inner membrane protein | -0.73 |
| b0849 | *grxA* | glutaredoxin 1, redox coenzyme for ribonucleotide reductase (RNR1a) | -0.42 |
| b0873 | *hcp* | hybrid-cluster | -0.79 |
| b1498 | *ydeN* | conserved protein | -0.65 |
| b1679 | *sufE* | sulfur acceptor protein | -3.55 |
| b1680 | *sufS* | cysteine desulfurase, stimulated by SufE; selenocysteine lyase, PLP-dependent | -2.86 |
| b1681 | *sufD* | FeS cluster assembly protein of SufBCD complex | -1.59 |
| b1682 | *sufC* | component of SufBCD complex, ATP-binding component of ABC superfamily | -1.30 |
| b1683 | *sufB* | component of SufBCD complex | -0.74 |
| b1684 | *sufA* | Fe-S cluster assembly protein | -0.47 |
| b2217 | *rcsB* | DNA-binding response regulator in two-component regulatory system with RcsC and YojN | -0.29 |
| b2218 | *rcsC* | hybrid sensory kinase in two-component regulatory system with RcsB and YojN | -0.43 |
| b2355 | *yfdl* | Predicted inner membrane protein | -0.11 |
| b4367 | *fhuF* | ferric iron reductase involved in ferric hydroximate transport | -2.14 |

Table S4. Expression of SoxRS regulon genes in the Trans10-*nfiS* strain

| Blattner number | Gene name | Functional description | Log_2_ ratio |
| --- | --- | --- | --- |
| Upregulated |  |  |  |
| b0376 | *ampH* | penicillin-binding protein | 1.31 |
| b0425 | *panE* | 2-dehydropantoate reductase, NADPH-specific | 1.17 |
| b0463 | *acrA* | multidrug efflux system | 1.64 |
| b0843 | *ybjH* | predicted protein | 2.49 |
| b0852 | *rimK* | ribosomal protein S6 modification protein | 1.39 |
| b0910 | *cmk* | cytidylate kinase | 1.26 |
| b0952 | *ymbA* | conserved protein | 1.16 |
| b1164 | *ycgZ* | connector protein for RcsB regulation of biofilm and acid-resistance | 1.23 |
| b1431 | *ydcL* | lipoprotein | 1.15 |
| b1451 | *yncD* | predicted iron outer membrane transporter | 1.14 |
| b1530 | *marR* | DNA-binding transcriptional repressor of multiple antibiotic resistance | 2.80 |
| b1531 | *marA* | DNA-binding transcriptional dual activator of multiple antibiotic resistance | 3.96 |
| b1532 | *marB* | predicted protein | 4.11 |
| b2962 | *yggX* | protein that protects iron-sulfur proteins against oxidative damage | 1.28 |
| b3035 | *tolC* | transport channel | 1.22 |
| b3546 | *eptB* | KDO phosphoethanolamine transferase, Ca^2+^-inducible | 2.08 |
| b4062 | *soxS* | DNA-binding transcriptional dual regulator | 0.94 |
| Downregulated | | | |
| b0124 | *gcd* | glucose dehydrogenase | -1.67 |
| b0156 | *erpA* | iron-sulfur cluster insertion protein | -1.17 |
| b0389 | *yaiA* | predicted protein | -3.50 |
| b0478 | *ybaL* | predicted transporter with NAD(P)-binding Rossmann-fold domain | -1.22 |
| b0603 | *ybdO* | predicted DNA-binding transcriptional regulator | -2.27 |
| b0762 | *ybhT* | predicted protein | -1.78 |
| b0829 | *gsiA* | glutathione transporter ATP-binding protein, ABC superfamily | -1.82 |
| b0864 | *artP* | arginine transporter subunit | -1.01 |
| b0871 | *poxB* | pyruvate dehydrogenase (pyruvate oxidase), thiamin-dependent, FAD-binding | -3.72 |
| b1052 | *b1052* | Z1687: hypothetical protein | -2.96 |
| b1053 | *mdtG* | predicted drug efflux system | -3.41 |
| b1167 | *ymgC* | predicted protein | -1.12 |
| b1168 | *ycgG* | predicted cyclic-di-GMP phosphodiesterase | -1.85 |
| b1377 | *ompN* | outer membrane pore protein N, non-specific | -1.11 |
| b3036 | *ygiA* | hypothetical protein | -1.06 |
| b3519 | *treF* | cytoplasmic trehalase | -2.24 |
| b3547 | *yhjX* | Inner membrane protein, predicted oxalate-formate antiporter | -1.34 |
| b3625 | *rfaY* | lipopolysaccharide core biosynthesis protein | -1.10 |
| b3909 | *kdgT* | 2-keto-3-deoxy-D-gluconate transporter | -1.15 |
| b4060 | *yjcB* | conserved protein | -3.36 |
| b4063 | *soxR* | DNA-binding transcriptional dual regulator, Fe-S center for redox-sensing | -0.24 |
| b4396 | *rob* | right *oriC*-binding transcriptional activator, AraC family | -2.70 |
| b4430 | *rydB* | *rydB*: ncRNA | -1.16 |

Table S5. Expression of Fur regulon genes in the Trans10-*nfiS* strain

| Blattner number | Gene name | Functional description | Log_2_ ratio |
| --- | --- | --- | --- |
| Upregulated | | | |
| b0150 | *fhuA* | ferrichrome outer membrane transporter | 1.19 |
| b0591 | *entS* | enterobactin exporter, iron-regulated | 1.20 |
| b0804 | *ybiX* | conserved protein, Fe (II)-dependent oxygenase superfamily | 1.97 |
| b1018 | *efeO* | inactive ferrous ion transporter EfeUOB | 1.55 |
| b1102 | *fhuE* | ferric-rhodotorulic acid outer membrane transporter | 2.59 |
| b1252 | *tonB* | membrane spanning protein in TonB-ExbB-ExbD transport complex | 1.07 |
| b1452 | *yncE* | conserved protein | 1.20 |
| b1495 | *yddB* | predicted porin protein | 3.85 |
| b1496 | *yddA* | fused predicted multidrug transporter subunits of ABC superfamily: membrane component/ATP-binding component | 2.18 |
| b2000 | *flu* | CP4-44 prophage; antigen 43 (Ag43) phase-variable biofilm formation autotransporter | 1.46 |
| b2155 | *cirA* | catecholate siderophore receptor CirA | 1.46 |
| b2673 | *nrdH* | Hydrogen donor for NrdEF electron transport system; glutaredoxin-like protein | 3.41 |
| b2674 | *nrdI* | flavodoxin required for NrdEF cluster assembly | 2.62 |
| b2676 | *nrdF* | ribonucleoside-diphosphate reductase 2, beta subunit, ferritin-like protein | 3.17 |
| b3070 | *yqjH* | predicted siderophore interacting protein | 1.34 |
| b4291 | *fecA* | KpLE2 phage-like element; ferric citrate outer membrane transporter | 1.88 |
| Downregulated | | | |
| b0590 | *fepD* | iron-enterobactin transporter subunit | -1.70 |
| b0597 | *entH* | thioesterase required for efficient enterobactin production | -1.13 |
| b1679 | *sufE* | sulfur acceptor protein | -3.55 |
| b1680 | *sufS* | cysteine desulfurase, stimulated by SufE; selenocysteine lyase, PLP-dependent | -2.86 |
| b1681 | *sufD* | FeS cluster assembly protein of SufBCD complex | -1.59 |
| b1682 | *sufC* | component of SufBCD complex, ATP-binding component of ABC superfamily | -1.30 |
| b1705 | *ydiE* | conserved protein, hemin uptake protein HemP homolog | -1.14 |
| b1995 | *yeoA* | putative outer membrane receptor for iron compound or colicin | -2.78 |
| b2524 | *iscX* | conserved protein | -1.18 |
| b2525 | *fdx* | [2Fe-2S] ferredoxin | -1.15 |
| b3336 | *bfr* | bacterioferritin, iron storage and detoxification protein | -1.92 |
| b4367 | *fhuF* | ferric iron reductase involved in ferric hydroximate transport | -2.14 |

Table S6. Expression level of Genes involved in cysteine synthesis system in the Trans10-*nfiS* strain

| Blattner number | Gene name | Functional description | Log_2_ ratio |
| --- | --- | --- | --- |
| b1729 | *ydjN* | predicted transporter | -1.77 |
| b2012 | *yeeD* | conserved protein | -4.02 |
| b2013 | *yeeE* | inner membrane protein, UPF0394 family | -3.15 |
| b2414 | *cysK* | cysteine synthase A, O-acetylserine sulfhydrolase A subunit | -2.29 |
| b2421 | *cysM* | cysteine synthase B (O-acetylserine sulfhydrolase B) | -2.63 |
| b2422 | *cysA* | sulfate/thiosulfate transporter subunit | -4.37 |
| b2423 | *cysW* | sulfate/thiosulfate transporter subunit | -4.23 |
| b2424 | *cysU* | sulfate/thiosulfate transporter subunit | -4.21 |
| b2425 | *cysP* | thiosulfate-binding protein | -3.46 |
| b2750 | *cysC* | adenosine 5'-phosphosulfate kinase | -3.88 |
| b2751 | *cysN* | sulfate adenylyltransferase, subunit 1 | -4.16 |
| b2752 | *cysD* | sulfate adenylyltransferase, subunit 2 | -4.52 |
| b2762 | *cysH* | 3'-phosphoadenosine 5'-phosphosulfate reductase | -4.86 |
| b2763 | *cysI* | sulfite reductase, beta subunit, NAD(P)-binding, heme-binding | -4.05 |
| b2764 | *cysJ* | sulfite reductase, alpha subunit, flavoprotein | -5.09 |
| b3607 | *cysE* | serine acetyltransferase | -0.66 |
| b3917 | *sbp* | sulfate transporter subunit | -5.21 |

Table S7. Expression level of Genes coded ribonucleotide reductases in the

Trans10-*nfiS* strain

| Blattner number | Gene name | Functional description | Log_2_ ratio |
| --- | --- | --- | --- |
| b2235 | *nrdB* | ribonucleoside diphosphate reductase 1; beta subunit; ferritin like protein | 1.76 |
| b2673 | *nrdH* | Hydrogen donor for NrdEF electron transport system; glutaredoxin-like protein | 3.41 |
| b2674 | *nrdI* | flavodoxin required for NrdEF cluster assembly | 2.62 |
| b2675 | *nrdE* | ribonucleoside-diphosphate reductase 2, alpha subunit | 0.84 |
| b2676 | *nrdF* | ribonucleoside-diphosphate reductase 2, beta subunit, ferritin-like protein | 3.17 |

Table S8. Expression level of Genes involved in biotin synthesis in the

Trans10-*nfiS* strain

| Blattner number | Gene name | Functional description | Log_2_ ratio |
| --- | --- | --- | --- |
| b0773 | *ybhB* | kinase inhibitor homolog, UPF0098 family | -0.57 |
| b0774 | *bioA* | 7,8-diaminopelargonic acid synthase, PLP-dependent | 1.34 |
| b0775 | *bioB* | biotin synthase | -0.85 |
| b0776 | *bioF* | 8-amino-7-oxononanoate synthase | -1.61 |
| b0777 | *bioC* | malonyl-CoA methyltransferase, SAM-dependent | -1.67 |
| b0778 | *bioD* | dethiobiotin synthetase | 0.34 |

Table S9. Expression level of Genes involved in acid resistance system in the

Trans10-*nfiS* strain

| Blattner number | Gene name | Functional description | Log_2_ ratio |
| --- | --- | --- | --- |
| b1492 | *gadC* | predicted glutamate: gamma-aminobutyric  acid antiporter | -2.80 |
| b1493 | *gadB* | glutamate decarboxylase B, PLP-dependent | -3.54 |
| b1499 | *ydeO* | transcriptional activator for *mdtEF* | -0.75 |
| b1500 | *safA* | Two component system connector membrane protein, EvgSA to PhoQP | -0.89 |
| b1501 | *ydeP* | predicted oxidoreductase | -0.38 |
| b2369 | *evgA* | DNA-binding response regulator in two-component regulatory system with EvgS | -2.30 |
| b3506 | *slp* | outer membrane lipoprotein | -2.24 |
| b3507 | *dctR* | predicted DNA-binding transcriptional regulator | -1.22 |
| b3508 | *yhiD* | predicted Mg^2+^ transport ATPase, inner membrane protein | -0.95 |
| b3509 | *hdeB* | acid-resistance protein | -1.46 |
| b3510 | *hdeA* | stress response protein acid-resistance protein | -1.63 |
| b3511 | *hdeD* | acid-resistance membrane protein | -1.80 |
| b3512 | *gadE* | DNA-binding transcriptional activator | -1.44 |
| b3513 | *mdtE* | multidrug resistance efflux transporter | -1.30 |
| b3514 | *mdtF* | multidrug transporter, RpoS-dependent | -0.45 |
| b3515 | *gadW* | transcriptional activator of *gadA* and *gadBC* in absence of GadX | -2.36 |
| b3516 | *gadX* | DNA-binding transcriptional dual regulator | -2.70 |
| b3517 | *gadA* | glutamate decarboxylase A, PLP-dependent | -3.53 |
| b4452 | *gadY* | *gadY*: ncRNA | -2.40 |

Table S10. IntaRNA prediction results

| Rank | P-value | Fdr value | Target | Position  mRNA^a^ | Position  NfiS | Energy  kcal/mol | Gene | Annotation |
| --- | --- | --- | --- | --- | --- | --- | --- | --- |
| 1 | 6.96E-05 | 0.15061 | b2358 | 204 -- 295 | 193 -- 252 | -34.3214 | *oweS* | pseudogene CPS-53 (KpLE1) prophage bacteriophage replication protein O familyPhage or Prophage Related |
| 2 | 6.97E-05 | 0.15061 | b3654 | 373 -- 432 | 200 -- 252 | -34.3173 | *xanP* | xanthine permease |
| 3 | 0.000503 | 0.55458 | b2536 | 496 -- 553 | 188 -- 233 | -30.8902 | *hcaT* | putative 3-phenylpropionic transporter |
| 4 | 0.000631 | 0.55458 | b3603 | 416 -- 474 | 194 -- 245 | -30.4701 | *lldP* | L-lactate permease |
| 5 | 0.000691 | 0.55458 | b2576 | 384 -- 418 | 217 -- 252 | -30.2999 | *srmB* | ATP-dependent RNA helicase |
| 6 | 0.000796 | 0.55458 | b2611 | 313 -- 372 | 188 -- 242 | -30.0325 | *ypjD* | cytochrome c assembly protein family inner membrane protein |
| 7 | 0.001176 | 0.55458 | b3783 | 524 -- 595 | 188 -- 254 | -29.2827 | *rho* | transcription termination factor |
| 8 | 0.001216 | 0.55458 | b3096 | 300 -- 391 | 157 -- 248 | -29.2184 | *mzrA* | modulator of EnvZ/OmpR regulon |
| 9 | 0.001227 | 0.55458 | b1061 | 166 -- 218 | 193 -- 250 | -29.2007 | *dinI* | DNA damage-inducible protein I |
| 10 | 0.001284 | 0.55458 | b3060 | 440 -- 491 | 199 -- 252 | -29.1117 | *ttdR* | transcriptional activator of *ttdABT* |
| 11 | 0.00153 | 0.56091 | b1125 | 472 -- 585 | 135 -- 253 | -28.7671 | *potB* | spermidine/putrescine ABC transporter permease |
| 12 | 0.001559 | 0.56091 | b0920 | 332 -- 390 | 200 -- 244 | -28.7311 | *elyC* | envelope biogenesis factor DUF218 superfamily protein |
| 13 | 0.00218 | 0.66788 | b2135 | 20 -- 45 | 228 -- 251 | -28.0602 | *yohC* | Yip1 family inner membrane protein |
| 14 | 0.00224 | 0.66788 | b2475 | 89 -- 147 | 193 -- 251 | -28.0057 | *ypfJ* | putative neutral zinc metallopeptidase |
| 15 | 0.00232 | 0.66788 | b0338 | 363 -- 458 | 158 -- 252 | -27.935 | *cynR* | transcriptional activator of *cyn* operon autorepressor |
| 16 | 0.002577 | 0.69558 | b3592 | 41 -- 70 | 224 -- 254 | -27.7211 | *yibF* | glutathione S-transferase homolog |
| 17 | 0.002936 | 0.746 | b3234 | 397 -- 430 | 218 -- 254 | -27.4535 | *degQ* | serine endoprotease periplasmic |
| 18 | 0.003379 | 0.81075 | b3946 | 225 -- 269 | 202 -- 252 | -27.1634 | *fsaB* | fructose-6-phosphate aldolase 2 |
| 19 | 0.003602 | 0.81879 | b3413 | 499 -- 599 | 156 -- 254 | -27.0304 | *gntX* | DNA catabolic protein |
| 20 | 0.003892 | 0.82496 | b0269 | 210 -- 265 | 188 -- 241 | -26.8684 | *yagF* | CP4-6 prophage dehydratase family protein |
| 21 | 0.004123 | 0.82496 | b0875 | 367 -- 515 | 127 -- 251 | -26.7479 | *aqpZ* | aquaporin Z |
| 22 | 0.004518 | 0.82496 | b3115 | 75 -- 168 | 151 -- 254 | -26.5548 | *tdcD* | propionate kinase/acetate kinase C anaerobic |
| 23 | 0.004603 | 0.82496 | b0089 | 193 -- 234 | 210 -- 251 | -26.5152 | *ftsW* | putative lipid II flippase integral membrane protein FtsZ ring stabilizer |
| 24 | 0.00487 | 0.82496 | b1859 | 401 -- 525 | 132 -- 250 | -26.3957 | *znuB* | zinc ABC transporter permease |
| 25 | 0.004885 | 0.82496 | b2832 | 320 -- 363 | 188 -- 236 | -26.3891 | *ygdQ* | UPF0053 family inner membrane protein |
| 26 | 0.005238 | 0.82496 | b1660 | 367 -- 459 | 159 -- 245 | -26.2408 | *ydhC* | putative arabinose efflux transporter |
| 27 | 0.005667 | 0.82496 | b2485 | 434 -- 478 | 196 -- 239 | -26.0721 | *hyfE* | hydrogenase 4 membrane subunit |
| 28 | 0.005935 | 0.82496 | b2877 | 38 -- 89 | 211 -- 254 | -25.9727 | *mocA* | CTP:molybdopterin cytidylyltransferase |
| 29 | 0.00611 | 0.82496 | b3935 | 315 -- 360 | 188 -- 238 | -25.9103 | *priA* | Primosome factor n' (replication factor Y) |
| 30 | 0.006117 | 0.82496 | b4702 | 534 -- 573 | 202 -- 233 | -25.9077 | *mgtL* | regulatory leader peptide for mgtA |
| 31 | 0.006352 | 0.82496 | b1580 | 529 -- 583 | 194 -- 251 | -25.8265 | *rspB* | putative Zn-dependent NAD(P)-binding oxidoreductase |
| 32 | 0.006795 | 0.82496 | b2633 | 295 -- 345 | 190 -- 236 | -25.6802 | *yfjQ* | CP4-57 prophage uncharacterized protein |
| 33 | 0.006863 | 0.82496 | b4078 | 346 -- 376 | 212 -- 243 | -25.6586 | *yjcO* | Sel1 family TPR-like repeat protein |
| 34 | 0.007253 | 0.82496 | b3235 | 419 -- 466 | 205 -- 250 | -25.5383 | *degS* | serine endoprotease periplasmic |
| 35 | 0.007404 | 0.82496 | b2820 | 440 -- 480 | 200 -- 239 | -25.4936 | *recB* | exonuclease V (RecBCD complex) beta subunit |
| 36 | 0.007405 | 0.82496 | b2380 | 435 -- 486 | 196 -- 244 | -25.4931 | *ypdA* | sensor kinase regulating yhjX pyruvate-responsive YpdAB two-component system |
| 37 | 0.007486 | 0.82496 | b3020 | 355 -- 390 | 215 -- 243 | -25.4693 | *ygiS* | putative ABC transporter permease |
| 38 | 0.008285 | 0.82496 | b3858 | 165 -- 201 | 205 -- 238 | -25.2472 | *yihD* | DUF1040 protein YihD |
| 39 | 0.008387 | 0.82496 | b2982 | 75 -- 147 | 193 -- 253 | -25.2203 | *insH1* | IS5 transposase and trans-activator |
| 40 | 0.008441 | 0.82496 | b1751 | 149 -- 196 | 201 -- 243 | -25.206 | *ydjY* | putative ferrodoxin-like lipoprotein |
| 41 | 0.008636 | 0.82496 | b2482 | 327 -- 366 | 200 -- 240 | -25.1557 | *hyfB* | hydrogenase 4 membrane subunit |
| 42 | 0.00869 | 0.82496 | b3771 | 527 -- 572 | 21 -- 68 | -25.142 | *ilvD* | dihydroxyacid dehydratase |
| 43 | 0.009045 | 0.82496 | b4242 | 340 -- 379 | 202 -- 233 | -25.0534 | *mgtA* | magnesium transporter |
| 44 | 0.009072 | 0.82496 | b0067 | 456 -- 513 | 193 -- 247 | -25.047 | *thiP* | thiamine/thiamine pyrophosphate ABC transporter permease |
| 45 | 0.009137 | 0.82496 | b4074 | 536 -- 588 | 205 -- 252 | -25.0311 | *nrfE* | heme lyase (NrfEFG) for insertion of heme into c552 subunit NrfE |
| 46 | 0.009156 | 0.82496 | b0597 | 203 -- 245 | 211 -- 254 | -25.0265 | *entH* | enterobactin synthesis proofreading thioesterase |
| 47 | 0.009571 | 0.82496 | b2985 | 562 -- 599 | 189 -- 228 | -24.9282 | *yghS* | putative ATP-binding protein |
| 48 | 0.009582 | 0.82496 | b0599 | 299 -- 355 | 193 -- 244 | -24.9256 | *ybdH* | putative oxidoreductase |
| 49 | 0.009766 | 0.82496 | b4557 | 300 -- 328 | 216 -- 243 | -24.8835 | *yidD* | membrane protein insertion efficiency factor UPF0161 family inner membrane protein |
| 50 | 0.00985 | 0.82496 | b4362 | 427 -- 468 | 203 -- 243 | -24.8644 | *dnaT* | DNA biosynthesis protein (primosomal protein I) |
| 51 | 0.00989 | 0.82496 | b2556 | 358 -- 404 | 206 -- 253 | -24.8553 | *glrK* | sensor protein kinase regulating glmY sRNA in two-component system with response regulator GlrR |
| 52 | 0.009932 | 0.82496 | b3998 | 50 -- 127 | 178 -- 253 | -24.8458 | *nfi* | endonuclease V deoxyinosine 3' endonuclease |
| 53 | 0.010202 | 0.83138 | b3667 | 394 -- 426 | 173 -- 205 | -24.7861 | *uhpC* | membrane protein regulates uhpT expression |
| 54 | 0.010398 | 0.83167 | b3571 | 297 -- 345 | 215 -- 252 | -24.7436 | *malS* | alpha-amylase |
| 55 | 0.01147 | 0.88719 | b3190 | 10 -- 29 | 192 -- 211 | -24.5238 | *ibaG* | acid stress protein putative BolA family transcriptional regulator |
| 56 | 0.011503 | 0.88719 | b0734 | 525 -- 593 | 174 -- 251 | -24.5173 | *cydB* | cytochrome d terminal oxidase subunit II |
| 57 | 0.011867 | 0.89584 | b3609 | 374 -- 397 | 228 -- 252 | -24.4473 | *secB* | protein export chaperone |
| 58 | 0.012066 | 0.89584 | b1709 | 547 -- 589 | 202 -- 242 | -24.4097 | *btuD* | vitamin B12 ABC transporter ATPase |
| 59 | 0.012762 | 0.89584 | b4032 | 14 -- 81 | 189 -- 236 | -24.2831 | *malG* | maltose transporter subunit |
| 60 | 0.012875 | 0.89584 | b3705 | 40 -- 68 | 216 -- 243 | -24.263 | *yidC* | membrane protein insertase |
| 61 | 0.013103 | 0.89584 | b0861 | 344 -- 444 | 158 -- 251 | -24.2232 | *artM* | arginine ABC transporter permease |
| 62 | 0.01315 | 0.89584 | b0833 | 18 -- 82 | 184 -- 244 | -24.2152 | *yliE* | putative membrane-anchored cyclic-di-GMP phosphodiesterase |
| 63 | 0.013603 | 0.89584 | b2661 | 453 -- 497 | 216 -- 252 | -24.1382 | *gabD* | succinate-semialdehyde dehydrogenase I NADP-dependent |
| 64 | 0.014027 | 0.89584 | b4198 | 222 -- 274 | 28 -- 81 | -24.0683 | *ulaF* | L-ribulose 5-phosphate 4-epimerase |
| 65 | 0.014087 | 0.89584 | b4230 | 90 -- 157 | 198 -- 251 | -24.0586 | *ytfT* | putative sugar ABC transporter permease |
| 66 | 0.014277 | 0.89584 | b1110 | 540 -- 599 | 8 -- 65 | -24.028 | *ycfJ* | uncharacterized protein |
| 67 | 0.01545 | 0.89584 | b2232 | 349 -- 471 | 137 -- 254 | -23.8473 | *ubiG* | bifunctional 3-demethylubiquinone-9 3-methyltransferase/ 2-octaprenyl-6-hydroxy phenol methylase |
| 68 | 0.015596 | 0.89584 | b3478 | 312 -- 363 | 188 -- 236 | -23.8258 | *nikC* | nickel ABC transporter permease |
| 69 | 0.01572 | 0.89584 | b3882 | 6 -- 68 | 193 -- 254 | -23.8075 | *yihU* | 3-sulpholactaldehyde (SLA) reductase NADH-dependent gamma-hydroxybutyrate dehydrogenase NADH-dependent |
| 70 | 0.015913 | 0.89584 | b0002 | 512 -- 565 | 205 -- 253 | -23.7795 | *thrA* | Bifunctional aspartokinase/homoserine dehydrogenase 1 |
| 71 | 0.015969 | 0.89584 | b1849 | 5 -- 64 | 195 -- 243 | -23.7714 | *purT* | phosphoribosylglycinamide formyltransferase 2 |
| 72 | 0.016123 | 0.89584 | b3393 | 326 -- 377 | 198 -- 253 | -23.7494 | *hofO* | DNA catabolic protein |
| 73 | 0.016247 | 0.89584 | b1117 | 247 -- 294 | 218 -- 252 | -23.7317 | *lolD* | outer membrane-specific lipoprotein transporter subunit |
| 74 | 0.016492 | 0.89584 | b0527 | 309 -- 334 | 227 -- 252 | -23.6973 | *ybcI* | DUF457 family inner membrane protein |
| 75 | 0.016637 | 0.89584 | b2896 | 332 -- 404 | 169 -- 225 | -23.6771 | *cptA* | toxin of CptAB toxin-antitoxin pair |
| 76 | 0.016714 | 0.89584 | b3191 | 463 -- 482 | 192 -- 211 | -23.6664 | *mlaB* | ABC transporter maintaining OM lipid asymmetry cytoplasmic STAS component |
| 77 | 0.016778 | 0.89584 | b3050 | 506 -- 581 | 167 -- 239 | -23.6576 | *yqiJ* | DUF1449 family inner membrane protein |
| 78 | 0.016824 | 0.89584 | b4080 | 214 -- 253 | 206 -- 244 | -23.6513 | *mdtP* | outer membrane factor of efflux pump |
| 79 | 0.016886 | 0.89584 | b2998 | 115 -- 144 | 220 -- 247 | -23.6428 | *yghW* | DUF2623 family protein |
| 80 | 0.016906 | 0.89584 | b1292 | 48 -- 108 | 199 -- 250 | -23.6401 | *sapC* | antimicrobial peptide transport ABC transporter permease |
| 81 | 0.01704 | 0.89584 | b4620 | 353 -- 408 | 198 -- 249 | -23.6218 | *yjbT* | putative periplasmic protein |
| 82 | 0.017064 | 0.89584 | b2196 | 394 -- 487 | 157 -- 248 | -23.6185 | *ccmF* | heme lyase CcmF subunit |
| 83 | 0.017316 | 0.89584 | b3433 | 391 -- 438 | 192 -- 241 | -23.5847 | *asd* | aspartate-semialdehyde dehydrogenase NAD(P)-binding |
| 84 | 0.017464 | 0.89584 | b1775 | 383 -- 432 | 167 -- 219 | -23.565 | *ydjK* | putative MFS sugar transporter membrane protein |
| 85 | 0.017651 | 0.89584 | b3018 | 416 -- 453 | 167 -- 209 | -23.5403 | *plsC* | 1-acyl-sn-glycerol-3-phosphate acyltransferase |
| 86 | 0.018041 | 0.89584 | b0503 | 435 -- 498 | 203 -- 249 | -23.4897 | *mnmH* | tRNA 2-selenouridine synthase selenophosphate-dependent |
| 87 | 0.018292 | 0.89584 | b0632 | 479 -- 522 | 209 -- 253 | -23.4576 | *dacA* | D-alanyl-D-alanine carboxypeptidase (penicillin-binding protein 5) |
| 88 | 0.018305 | 0.89584 | b0166 | 16 -- 82 | 199 -- 253 | -23.4559 | *dapD* | 2345-tetrahydropyridine-2-carboxylate N-succinyltransferase |
| 89 | 0.018812 | 0.89584 | b1623 | 505 -- 576 | 167 -- 240 | -23.3924 | *add* | adenosine deaminase |
| 90 | 0.018926 | 0.89584 | b2254 | 1 -- 35 | 201 -- 229 | -23.3784 | *arnC* | undecaprenyl phosphate-L-Ara4FN transferase |
| 91 | 0.019047 | 0.89584 | b2164 | 386 -- 415 | 8 -- 38 | -23.3635 | *psuT* | putative nucleoside transporter |
| 92 | 0.019168 | 0.89584 | b2558 | 340 -- 371 | 169 -- 211 | -23.3488 | *mltF* | membrane-bound lytic transglycosylase F murein hydrolase |
| 93 | 0.01929 | 0.89584 | b3039 | 92 -- 154 | 203 -- 252 | -23.334 | *ygiD* | 45- DOPA-extradiol-dioxygenase |
| 94 | 0.019797 | 0.90959 | b2424 | 373 -- 412 | 216 -- 252 | -23.2735 | *cysU* | sulfate/thiosulfate ABC transporter permease |
| 95 | 0.020496 | 0.92006 | b2555 | 324 -- 372 | 200 -- 254 | -23.1923 | *yfhG* | putative outer membrane protein modulating the QseEF response |
| 96 | 0.020556 | 0.92006 | b0567 | 100 -- 140 | 27 -- 64 | -23.1855 | *ybcH* | PRK09936 family protein |
| 97 | 0.020677 | 0.92006 | b3080 | 323 -- 375 | 169 -- 218 | -23.1717 | *ygjK* | alpha-glucosidase |
| 98 | 0.020952 | 0.92006 | b0496 | 322 -- 378 | 203 -- 252 | -23.1408 | *ybbP* | putative ABC transporter permease |
| 99 | 0.02109 | 0.92006 | b0298 | 105 -- 127 | 205 -- 229 | -23.1254 | *insE1* | IS3 transposase A |
| 100 | 0.023778 | 0.96305 | b1799 | 317 -- 368 | 202 -- 252 | -22.8427 | *dmlR* | transcriptional activator of *dmlA* |

^a^ The position of +1 of candidate target is 300 nt upstream of the start codon.
